# Sleep Disordered Breathing, Fatigue, and Sleepiness in HIV-Infected and -Uninfected Men

**DOI:** 10.1371/journal.pone.0099258

**Published:** 2014-07-03

**Authors:** Susheel P. Patil, Todd T. Brown, Lisa P. Jacobson, Joseph B. Margolick, Alison Laffan, Lisette Johnson-Hill, Rebecca Godfrey, Jacquett Johnson, Sandra Reynolds, Alan R. Schwartz, Philip L. Smith

**Affiliations:** 1 Division of Pulmonary and Critical Care Medicine, Johns Hopkins School of Medicine, Baltimore, Maryland, United States of America; 2 Division of Endocrinology and Metabolism, Johns Hopkins School of Medicine, Baltimore, Maryland, United States of America; 3 Department of Epidemiology, Johns Hopkins Bloomberg School of Public Health, Baltimore, Maryland, United States of America; 4 Department of Molecular Microbiology and Immunology, Johns Hopkins Bloomberg School of Public Health, Baltimore, Maryland, United States of America; University of Kansas Medical Center, United States of America

## Abstract

**Study Objectives:**

We investigated the association of HIV infection and highly active antiretroviral therapy (HAART) with sleep disordered breathing (SDB), fatigue, and sleepiness.

**Methods:**

HIV-uninfected men (HIV−; n = 60), HIV-infected men using HAART (HIV+/HAART+; n = 58), and HIV-infected men not using HAART (HIV+/HAART−; n = 41) recruited from two sites of the Multicenter AIDS cohort study (MACS) underwent a nocturnal sleep study, anthropometric assessment, and questionnaires for fatigue and the Epworth Sleepiness Scale. The prevalence of SDB in HIV- men was compared to that in men matched from the Sleep Heart Health Study (SHHS).

**Results:**

The prevalence of SDB was unexpectedly high in all groups: 86.7% for HIV−, 70.7% for HIV+/HAART+, and 73.2% for HIV+/HAART−, despite lower body-mass indices (BMI) in HIV+ groups. The higher prevalence in the HIV− men was significant in univariate analyses but not after adjustment for BMI and other variables. SDB was significantly more common in HIV− men in this study than those in SHHS, and was common in participants with BMIs <25 kg/m^2^. HIV+ men reported fatigue more frequently than HIV− men (25.5% vs. 6.7%; p = 0.003), but self-reported sleepiness did not differ among the three groups. Sleepiness, but not fatigue, was significantly associated with SDB.

**Conclusions:**

SDB was highly prevalent in HIV− and HIV+ men, despite a normal or slightly elevated BMI. The high rate of SDB in men who have sex with men deserves further investigation. Sleepiness, but not fatigue, was related to the presence of SDB. Clinicians caring for HIV-infected patients should distinguish between fatigue and sleepiness when considering those at risk for SDB, especially in non-obese men.

## Introduction

Sleep disordered breathing (SDB) is characterized by recurrent collapse of the upper airway, resulting in intermittent hypoxemia and brief arousals from sleep. Thus, SDB leads to poor sleep quality, daytime sleepiness, and impaired quality of life and cognitive function [1]–[3]. Although patients with SDB most commonly report excessive sleepiness as their primary symptom, some report fatigue [4], [5].

In patients with HIV infection, fatigue and sleepiness can impair quality of life and functional status [6]–[12] and may also be exacerbated by highly active antiretroviral therapy (HAART) [13]–[15]. HIV infection and HAART have been linked with SDB [13]–[16]. For example, HIV-infected patients receiving HAART who developed significant weight gain and lipodystrophy with progressive symptoms of snoring, fatigue, and daytime sleepiness were confirmed to have SDB [13]–[15]. Furthermore, HAART can induce adverse metabolic and morphologic changes which have been associated with SDB, including lipodystrophy (subcutaneous lipoatrophy and visceral obesity), insulin resistance, hypertension, and hyperlipidemia [13], [17]–[20]. Moreover, both HIV infection and HAART-induced increases in visceral adiposity may increase somnogenic cytokines [21], [22] and impair upper airway neural control, which in turn may exacerbate SDB [23], [24]. Thus, HIV infection and HAART could have independent and additive effects on SDB and fatigue. The relationship between HIV infection and sleepiness has been less well studied.

The overall objectives of the present study were to determine: a) whether SDB, fatigue, and sleepiness were more common in HIV-infected men than in HIV-uninfected men, b) whether HAART was associated with an increased prevalence and severity of SDB, and c) whether SDB was associated with fatigue and daytime sleepiness. To address these objectives, three groups of men were recruited from a longitudinal cohort study: a) HIV-uninfected men (HIV−), b) HIV-infected men using HAART (HIV+/HAART+), and c) HIV-infected men not using HAART (HIV+/HAART−). We hypothesized that HIV+/HAART+ men would have a greater prevalence of SDB, fatigue, and sleepiness than HIV+/HAART− and HIV− men, and that fatigue and sleepiness in HIV-infected men regardless of HAART status would be associated with SDB.

## Methods

### Study Sample and Recruitment

A cross-sectional study was nested within the Baltimore and Pittsburgh sites of the Multicenter AIDS Cohort Study (MACS), an ongoing prospective study of the natural and treated histories of HIV-1 infection in homosexual and bisexual men in which participants have been followed semiannually since 1984 [25]. From 2005–2008, three groups of men were invited by mailed letter to participate in the study: all HIV-infected men not using HAART (HIV+/HAART−), and random samples of HIV-infected men using HAART (HIV+/HAART+) and HIV-uninfected (HIV−) men of similar age and race. Men were considered HAART− if they had not used HAART for at least the past year. HAART was defined by the DHHS/Kaiser Guidelines [26], [27]. Potential participants were excluded for: a) history of upper airway surgery, b) use of supplemental oxygen, c) renal failure on dialysis, d) history of cirrhosis, e) unstable cardiovascular disease or uncontrolled hypertension in the previous 3 months, f) history of a psychiatric disorder severe enough to interfere with neuropsychological testing, and g) acute infectious central nervous system disease. The study was approved by the institutional review boards at the Johns Hopkins Bloomberg School of Public Health and the University of Pittsburgh Graduate School of Public Health, and all study participants provided written informed consent.

### Study Protocol

Men were admitted to the Johns Hopkins Bayview Clinical Research Unit (CRU) between 6–8 PM and completed the Epworth Sleepiness Scale (ESS), a measure of subjective sleepiness, and questions about fatigue symptoms (see below). A polysomnography study to assess sleep and breathing was conducted between 11 PM and 7 AM. Anthropometric measurements (neck, waist, and hip circumferences) were obtained either by direct measurement after the sleep study or from the nearest MACS study visit (the median interval between the MACS visit and the sleep study was 4 days, with an inter-quartile range of 0–39 days), as described below. Participants were discharged from the CRU after completion of the sleep study the following morning.

Additional data and specimens collected at semi-annual visits of the MACS were also analyzed: plasma HIV RNA quantification (RT-PCR; Amplicor HIV Monitor Assay, Roche Diagnostics, Nutley, NJ), T-cell subsets determined by flow cytometry [28], a standardized assessment of fat distribution (see below for further details) [29], and a medical history.

### Study Procedures

#### Polysomnography Testing

Participants were evaluated by full montage, in-laboratory nocturnal polysomnography (PSG). Continuous polygraphic recordings (EMBLA; Broomfield, CO) of a modified electrocardiographic (V6) lead, right and left electro-oculographic leads, submental and bilateral anterior tibialis surface electromyograms, and two electroencephalographic leads (C3-A2, O1-A2) were performed. Respiration was monitored throughout the test by a nasal pressure transducer (EMBLA) and oronasal thermocouples (Dymedix, Shoreview, MN), and with thoracic and abdominal inductive plethysmography (EMBLA). Continuous recording of the oxyhemoglobin saturation (SaO_2_) was obtained with an oximeter (XPOD; Nonin, Plymouth, MN).

Sleep study scoring was performed by a registered polysomnographic technologist and reviewed by a Diplomate in Sleep Medicine (A.R.S.). Sleep staging was performed on 30-second intervals according to the criteria of American Academy of Sleep Medicine (AASM) [30]. SDB events were scored based on the AASM 2007 Scoring Manual [30]. Hypopneas were scored using both the alternative (i.e., a ≥50% reduction in airflow lasting ≥10 seconds and associated with an EEG arousal or a decrease in SaO_2_ of ≥3%) and the recommended definitions (i.e., a ≥30% reduction in airflow lasting ≥10 seconds and associated a decrease in SaO_2_ of ≥4%). The alternative definition was preferred because, unlike the recommended definition, it incorporates both electroencephalographic (EEG) arousals and hypoxemia, each of which is an important contributor to sleepiness and fatigue [31]–[34]. The sum of apneas and hypopneas per hour of total sleep time utilizing the alternative definition was termed the respiratory disturbance index (RDI). SDB was defined as an RDI ≥5 events/hr. Cutpoints of ≥10 or ≥15 events/h were also evaluated. The severity of SDB was defined as mild (RDI 5.0–14.9 events/h), moderate (RDI 15.0–29.9 events/h), or severe (RDI ≥30.0 events/h), based on consensus criteria [35]. Results obtained using the recommended definition of SDB were also analyzed and yielded inferences almost identical to those obtained using the alternative definition. Therefore, results obtained using the recommended definition are presented in supplementary tables (Tables S2–S5). The sum of apneas and hypopneas per hour of total sleep time utilizing the recommended definition was termed the apnea-hypopnea index (AHI), and similar thresholds were used for defining SDB (i.e., AHI ≥5, ≥10, ≥15 events/hr) and disease severity. Apneas and hypopneas were classified as obstructive, central, or mixed according to AASM criteria [36].

#### Assessment of Sleepiness and Fatigue

The Epworth Sleepiness Scale (ESS) was used as a measure of subjective sleepiness, with a score of ≥11 indicating excessive daytime sleepiness [37]. The two questions in the 6-month MACS study visit core protocol regarding fatigue were: “Since your last visit, have you had persistent fatigue (feeling tired all the time) for at least 3 consecutive days”, and, for participants who responded “yes”, “Did that [fatigue] last for 2 weeks or longer?”.

#### Anthropometric Measurements

Height was measured to the nearest 0.5 cm using a stadiometer (Holtain; Wales, UK). Body weight was measured to the nearest 0.1 kg. Body mass index (BMI) was calculated as weight (kg)/[height (m)]^2^. Body circumferences were measured with a tape measure, with the participant standing in a relaxed position, at the waist (at the iliac crest at end expiration), hip (at the maximum extension of the buttocks), and neck (inferior to the laryngeal prominence and perpendicular to the long axis of the neck, with the patient seated and the head in the Frankfurt horizontal plane), using the Third National Health and Nutrition Examination Survey (NHANES III) protocol [38]. Body fat distribution was assessed by trained clinical staff at each MACS site, as previously described [29]. Lipohypertrophy was defined as the presence of moderate to severe fat accumulation in one or more compartments that included the abdomen, back of the neck, or the breasts; lipoatrophy was defined as the presence of moderate to severe fat wasting in one or more of the following compartments: the face, arms, legs, or buttocks.

### Statistical Analyses

The prevalence of SDB, fatigue, and sleepiness was examined first with comparisons of HIV− to HIV+ men, and then by comparisons between HIV+/HAART+ and HIV+/HAART− men. Fisher’s exact tests and analyses of variance/t-tests were used to compare categorical and continuous variables, respectively, among the groups, with results presented as means ± standard deviation (SD) or percent, unless otherwise stated. Non-parametric testing (e.g., Wilcoxon rank-sum, Kruskal-Wallis) was applied when data were not normally distributed, and results are presented as medians with inter-quartile ranges. Logistic regression models were used to examine the independent association of HIV and HAART status, age, and BMI with the presence of SDB. Separate models were constructed for each definition of SDB, using the different cutpoints described above. Logistic regression modeling was also used to examine the relation between SDB, fatigue, and sleepiness. The relation between self-reported sleepiness and fatigue was examined use chi-square testing and analyses of variance.

The potential effect of HAART on the prevalences of SDB, fatigue, and sleepiness was also examined by separating the HIV+/HAART− group into two groups: HIV+ with prior HAART use and HIV+ with no prior HAART use. Comparisons between these 2 groups and the HIV− men and HIV+/HAART+ men demonstrated similar overall conclusions; therefore, comparisons of the HIV− to HIV+ men and of the HIV+/HAART+ to HIV+/HAART− men are presented for ease of interpretation.

An analysis was conducted comparing the HIV− men to a subset of community-dwelling men from the Sleep Heart Health Study (SHHS), a study of the natural history of sleep apnea and its association with cardiovascular disease. SHHS participants were one-to-one matched to HIV− men on age (±3.5 years), sex, race, and BMI (±3 kg/m^2^). Fifty-eight of the 60 HIV− men in this study were successfully matched with men from SHHS. Comparisons of SDB prevalence and severity were performed using the AASM-recommended definition since this was the definition of SDB used in SHHS. All analyses were conducted using STATA 9.2 (Stata, College Station, TX).

## Results

### Participant Characteristics

One hundred fifty-nine men were enrolled: 60 were HIV− and 99 were HIV+. Of the latter, 58 (59%) were receiving HAART (HIV+/HAART+ group) and 41 (41%) were not (HIV+/HAART−). Their descriptive characteristics are shown in Table 1. The majority of each group was African-American, with similar percentages in the three groups. The HIV− men were slightly older and had higher BMIs, body circumferences and CD4 cell counts than the HIV+ men, and these differences were significant. Among the HIV+ groups, HIV+/HAART+ men had lower HIV viral loads and higher CD4 cell counts than HIV+/HAART− men, as expected, and also higher waist-to-hip ratios and likelihood of lipoatrophy. The prevalence of lipohypertrophy was similar among the three groups, as were most of the other medical and demographic variables analyzed. Because BMI and body circumference, which are major determinants of SDB, were higher in the HIV− men than the HIV+ men, adjustments, stratification, and matching strategies were employed in comparing SDB by HIV status; both unadjusted and adjusted results are presented.

**Table 1:** Participant Characteristics by HIV and HAART status.

	HIV−	All HIV+	P-value^a^	HIV+/HAART+	HIV+/HAART−	P-value^b^
	(n = 60)	(n = 99)		(n = 58)	(n = 41)	
Age, years	53.7 (9.3)	49.9 (8.1)	0.008	50.7 (7.9)	48.9 (8.4)*	0.29
Age (% >50 years old)	60.0	46.5	0.10	51.7	39.0*	0.15
Education, years	15.4 (3.3)	14.8 (2.7)	0.28	15.2 (2.5)	14.3 (3.0)	0.14
Race						
% Caucasian	41.7	44.4	0.73	48.3	39.0	0.42
% African American	58.3	55.6		51.7	61.0	
HIV disease status						
Plasma HIV RNA, copies/ml	–	<40 (<40–10,900)	–	<40 (<40–<40) †	15,000 (805–67,200)	0.0001
CD4 cell count, cells/mm^3^	993(790–1220)	531 (339–686)*	0.001	565 (382–686)*	428 (336–663)*	0.0001
Duration of HIV infection, yr	–	4.6 (3.3–18.4)	–	9.5 (3.6–18.9)	4.0 (3.1–12.3)	
History of AIDS, %	–	12.2%	–	17.2	4.9	0.12
HAART Regimen Base						
PI (%)	–	33.7	–	52.6	–	–
NNRTI (%)	–	23.5	–	40.3	–	–
NRTI (%)	–	3.1	–	5.3	–	–
Years from HAART initiation	–	8.8 (5.7–9.5)	–	8.8 (5.7–9.5)	–	–
Hypertension, %	56.1	43.8	0.18	52.7	31.7*	0.06
Diabetes Mellitus, %	22.7	10.5	0.11	12.5	7.4	0.69
Hepatitis B, %	1.7	4.0	0.65	3.4	4.9	1.00
Hepatitis C, %	21.7	27.3	0.43	20.7	36.6	0.11
Smoking						
Never, %	18.6	23.2	0.77	28.1	15.8	0.27
Ex-smoker, %	45.8	45.3		45.6	44.7	
Current smoker, %	35.6	31.6		26.3	39.5	
>3 alcoholic drinks/week, %	23.7	25.3	0.49	22.8	29.0	0.63
Illicit drug use^c^, %	40.0	38.4	0.87	32.8	46.3	0.39
Body-mass index, kg/m^2^	28.6 (7.2)	25.4 (4.3)	0.0006	25.5 (4.5)*	25.4 (4.1)*	0.92
Obese (% >30 kg/m^2^)	33.3	12.1	0.002	12.1*	12.2*	1.00
Regional Body Composition						
Neck circumference, cm	40.8 (3.5)^A^	39.5 (2.9)	0.01	39.9 (3.2)	39.0 (2.3)*	0.14
Waist circumference, cm	98.6 (16.9)	93.0 (12.1)	0.02	93.8 (11.5)	91.8 (12.8)*	0.42
Hip circumference, cm	102.3 (11.9)	95.7 (8.7)	0.0001	95.1 (8.1)*	96.5 (9.6)*	0.45
Waist:Hip Ratio	0.96 (0.08)	0.97 (0.07)	0.36	0.99 (0.06) † *	0.95 (0.06)	0.03
Observer-Assessed Lipodystrophy						
Lipoatrophy, n (%)	3 (5.0)	22 (22.2)	0.003	17 (29.3)* †	5 (12.2)	0.05
Lipohypertrophy, n(%)	23 (38.3)	31 (31.3)	0.391	19 (32.8)	12 (29.3)	0.83

Means (SD), median (25^th^ –75^th^ percentile), or proportions.

PI = protease inhibitor; NNRTI = non-nucleoside reverse transcriptase inhibitor; NRTI = nucleoside reverse transcriptase inhibitor.

a P-values are for comparison of HIV− to HIV+ participants.

b P-values are for comparison of HIV+/HAART+ to HIV+/HAART− participants.

c Illicit drug use was defined as any use of marijuana, nitrite inhalants, crack or cocaine, stimulants, or other illicit drugs since the last MACS visit.

*P < 0.05, compared to HIV−.

† P < 0.05, HIV+/HAART+ compared to HIV+/HAART−.

Comparisons of data represented with means were performed using t-tests for 2-group comparisons and linear regression with robust estimation of the standard errors. Comparisons of data represented by medians were performed using the Wilcoxon ranksum test for 2-group comparisons. Comparisons of categorical data represented by percent were performed using chi-square analysis and Fisher’s exact test.

### Prevalence and Severity of Sleep Disordered Breathing

The prevalence of SDB, defined as an RDI ≥5 events/h, was high: 87% in the HIV− men and 72% in the HIV+ men (Table 2, part A; p = 0.03). These prevalences were higher than those reported for community samples [1], [39]. Distributions of SDB severity were also different, with HIV− men having more severe SDB than HIV+ men (Table 2A; p = 0.03). When SDB was defined using higher thresholds of RDI (Table 2, part A), the prevalences were lower for both HIV− and HIV+ men and differences by HIV status were no longer significant. When SDB was defined using the AHI (see Table S1) or higher thresholds of AHI (Table S1, part A), the prevalence was lower overall (54–70%), as expected, but was slightly higher in HIV− than HIV+ men (p = 0.07). HIV+/HAART+ men and HIV+/HAART− men had similar SDB prevalences (70.7% and 73.2%, respectively; p = 0.83), and this was true for all SDB definitions used (Table 2A and Table S1, part A). The effects of the higher BMI in HIV− men relative to HIV+ men on SDB prevalence were further evaluated in the stratified and multivariable models shown below.

**Table 2:** Prevalence and Severity of Sleep Disordered Breathing (SDB) stratified by HIV status and HAART use.

	HIV –	All HIV+	P-value^a^	HIV+/HAART+	HIV+/HAART−	P-value^b^
	(N = 60)	(N = 99)		(N = 58)	(N = 41)	
**A. Prevalence**						
Prevalence By Respiratory Disturbance Index (RDI)						
RDI ≥5 events/h	52 (86.7)	71 (71.7)	0.03	41 (70.7)*	30 (73.2)	0.83
RDI ≥10 events/h	41 (68.3)	56 (56.6)	0.18	34 (58.6)	22 (53.7)	1.00
RDI ≥15 events/h	35 (58.3)	46 (46.5)	0.19	28 (48.3)	18 (43.9)	0.84
Prevalence By Severity (events/h)						
Normal (<5.0)	8 (13.3)	28 (28.3)	0.03	17 (29.3)	11 (30.6)	0.13
Mild (5.0–14.9)	17 (28.3)	25 (25.3)		13 (22.4)	12 (28.6)	
Moderate (15.0–29.9)	19 (31.7)	35 (35.4)		22 (37.9)	13 (31.7)	
Severe (≥30.0)	16 (26.7)	11 (11.1)		6 (10.3)	5 (12.2)	
**B. RDI and Respiratory Arousal Index Distribution**						
RDI (events/h)^c^						
NREM	18.1 (5.4–30.5)	11.2(3.8–21.6)	0.06	11.7 (3.8–21.6)	10.2 (3.8–21.2)	0.95
REM	19.3 (8.6–35.3)	14.0 (5.9–31.6)	0.18	13.3 (4.7–31.6)	15.9 (7.5–28.3)	0.67
Total	21.0 (7.9–30.9)	11.9(4.1–24.1)	0.02	12.1* (4.1–23.6)	11.9* (4.7–24.1)	0.97
Respiratory Arousal Index^d^ (events/h)						
NREM	11.9 (3.4–23.1)	6.2(1.9–14.6)	0.02	6.8 (2.3–15.9)	5.3* (1.8–12.1)	0.67
REM	6.8 (3.6–15.4)	7.0(2.4–17.2)	0.40	7.0 (2.3–17.4)	7.4 (2.6–13.6)	0.99
Total	12.0 (4.2–21.3)	6.1(2.7–15.3)	0.02	7.4 (3.1–16.3)	5.6* (2.5–14.2)	0.56

Values shown are N(%) or median (25^th^ percentile –75^th^ percentile).

a P-values are for comparison of HIV− to HIV+ participants.

b P-values are for comparison of HIV+/HAART+ to HIV+/HAART− participants.

c RDI is defined as the number of apneas and hypopneas (a ≥ 3% desaturation or an arousal) per hour of sleep.

d The respiratory arousal index is the number of arousals associated with an apnea or hypopnea per hour of sleep.

*P < 0.05, compared to HIV−.

Comparisons of data represented by medians were performed using the Wilcoxon ranksum test for 2 group comparisons. Comparisons of categorical data represented by percent were performed using chi-square analysis and the Fisher’s exact test.

**Figure 1:**
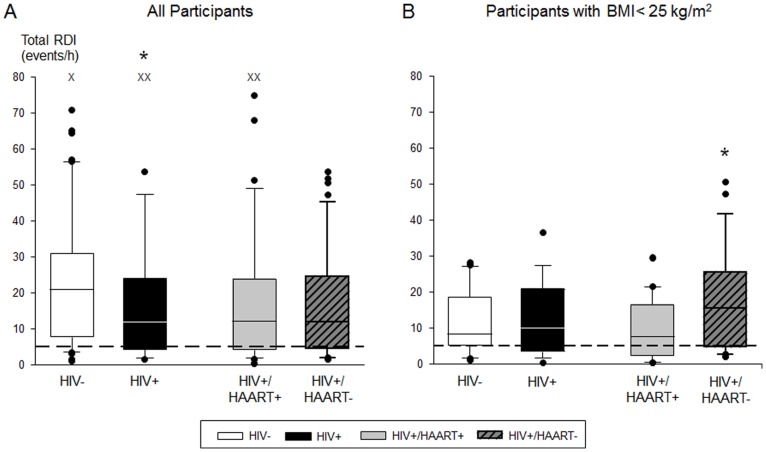
Severity of sleep disordered breathing for all study participants and study participants with BMI <25 kg/m^2^. Boxplots of RDI severity (median; 25^th^–75^th^ percentile) stratified by HIV infection and HAART status for A) all study participants and B) study participants with BMI <25 kg/m^2^. Among all participants (panel A), HIV− men (21.0; 7.9–30.9 events/h) had higher RDIs than HIV+men (11.9; 4.1–24.1 events/h; * indicates p = 0.02). In men with BMI <25 kg/m^2^ (panel B), HIV+/HAART− men (15.5; 4.7–25.4 events/h) had higher RDIs than HIV+/HAART+ men (7.7; 2.4–15.0 events/h; * indicates p = 0.02). The dashed line represents an RDI of 5 events/h. Boxes depict the 25^th^, 50^th^, and 75^th^ percentiles of the distributions; whiskers represent the 5^th^ and 95^th^ percentiles, and outliers are represented by solid circles beyond the whiskers. Outliers with an RDI >80 events/h are represented by an X (HIV− group: RDI 120.8 events/h; HIV+/HAART+ group: 86.5 and 88.5 events/h).

Table 2, part B and figure 1A show the distributions of RDI and the respiratory arousal index in the study groups during NREM, REM, and total sleep. RDIs were similar in NREM and REM sleep. The total RDI and respiratory arousal index were higher in the HIV− men than the HIV+ men (p = 0.02 for total RDI and total respiratory arousal index). In contrast, no significant differences in the RDI or the respiratory arousal index were observed between the HIV+/HAART+ and HIV+/HAART. Apneas and hypopneas were classified as obstructive (84% ±22%), central (11% ±20%), or mixed (5% ±10%) according to AASM criteria [36], with no significant differences between groups (p>0.10). Approximately 60% of respiratory events were associated with arousal in all groups. Identical inferences were obtained when using the AHI based on the AASM-recommended definition (Table S1, part B).

**Figure 2:**
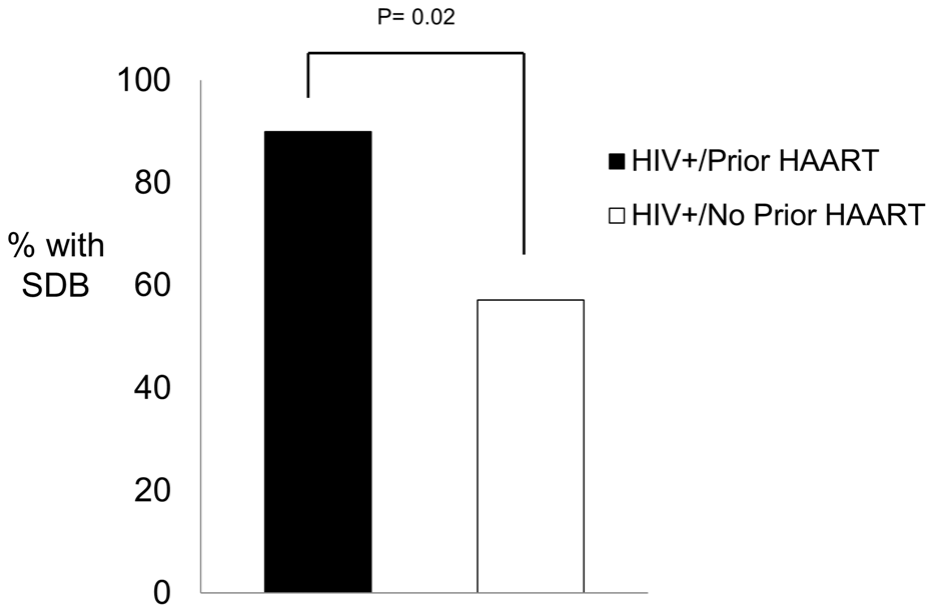
Prevalence of sleep disordered breathing by HAART exposure status. The HIV+/HAART− men were stratified by prior HAART (but had not taken HAART in the year prior to study enrollment) vs. no prior HAART exposure. Men with prior HAART exposure had a significantly higher prevalence of SDB than those that had no prior HAART exposure (90.0% vs. 57.1%, respectively; p = 0.02), despite a lower BMI.

Since approximately half of the HIV+/HAART− group had been exposed to HAART in the remote (>1 year) past, we examined whether men with (n = 20; BMI: 24.7±3.0 kg/m^2^) and without (n = 21; BMI: 26.0±5.0 kg/m^2^; p = 0.34) previous HAART exposure differed in their prevalence of SDB (figure 2). Despite a lower BMI, men with prior HAART exposure had a significantly higher prevalence of SDB than men without prior HAART exposure (90.0% vs. 57.1%, respectively; p = 0.02). The same relation was observed when using the AASM recommended definition of SDB (75.0% vs. 33.3%, respectively; p = 0.007).

### Comparison of HIV− Men to Community-Dwelling Men in the Sleep Heart Health Study (SHHS)

Since the prevalence of SDB in both HIV− and HIV+ men was higher than what has been reported in community samples [1], [39], we compared the HIV− men in the present study to a matched group of men in the general community, obtained from the Sleep Heart Health Study (SHHS). The two groups had similar BMI (28.3±5.9 vs. 28.3±5.5 kg/m^2^) and age (53.8±9.4 vs. 54.1±9.2 years old). However, the AHI (using the AASM recommended definition) and the oxygen desaturation index (ODI; desaturation events ≥4%/h) differed between the groups. Specifically, compared to the SHHS men the HIV− men in the present study had a higher prevalence of SDB (69.0% vs. 43.1%; p = 0.005), a higher median [25^th^–75^th^ percentile] AHI (7.2 [3.3–22.4] vs. 3.5 [1.5–13.9] events/h, respectively; p = 0.007), and a higher median ODI (10.2 [3.7–24.7] vs. 3.6 [1.0–12.6] events/h, respectively; p = 0.02). These data indicate that both the HIV− men and HIV+ men in this study were at higher risk for SDB than a sample of community-dwelling men.

### Relationship between SDB and Obesity, Age, Race, HIV status, and HAART use

In unadjusted analyses (Tables 3 and Table S2, left column), BMI and age were significantly associated with a higher prevalence of SDB, and being HIV+ was associated with a lower prevalence of SDB. Race, however, was not associated with SDB status. The prevalence of SDB was 77% in Caucasians compared to 73% in African Americans (p = 0.62). In addition, the median severity of SDB was 16 events/h in Caucasians compared to 11 events/h in African Americans (p = 0.40). African Americans were of similar BMI as Caucasians (26.2 [SD: 5.7] vs. 27.0 [SD: 5.8] kg/m2; p = 0.38). In multivariate analyses that adjusted for race, BMI, age, and HIV status, BMI and age remained significantly associated with the prevalence of SDB, but HIV status and HAART use did not. The effects of hypertension, diabetes mellitus, and smoking status on SDB status were also examined in view of the known associations of these conditions with SDB; none of these factors were significantly associated with SDB in univariate and multivariate logistic regression models (data not shown). Thus, differences in BMI and age very likely accounted, in large part, for the higher prevalence of SDB in the HIV−.

**Table 3:** Association of SDB with HIV status, BMI, age, and race.

	Unadjusted	Adjusted
HIV+*	**0.39 (0.16, 0.92)**	0.62 (0.25, 1.57)
BMI (kg/m^2^)	**1.16 (1.05,1.28)**	**1.14 (1.03, 1.27)**
Age (per decade)	**1.69 (1.06, 2.67)**	**1.74 (1.02, 2.97)**
Race†	1.06 (0.50, 2.23)	1.35(0.56, 3.28)

Similar results were obtained modeling an RDI ≥10 and an RDI ≥15 events/h.

Odd ratio (95% confidence interval).

*Reference group: HIV−;

† Reference group: Caucasian.

Note: Results did not change appreciably when comparing HIV+/HAART+ (unadjusted OR: 0.37 [95% CI: 0.15–0.94]; adjusted OR: 0.57 [95% CI: 0.21–1.54]) and HIV+/HAART− men(unadjusted OR: 0.42 [95% CI: 0.15–0.1.16]; adjusted OR: 0.71 [95% CI: 0.24–2.12]) to HIV− men.

To address the influence of BMI on SDB in HIV− and HIV+ men, two further anlayses were conducted. First, we examined the prevalence of SDB in men with BMI <25 kg/m^2^ (n = 71/159 [44.7%]). Again, the prevalence of SDB was quite high in both the HIV− and HIV+ men (75.0% and 62.8% respectively; p = 0.41; Table 4), and the differences were not significant. Although the severity of SDB was similar for HIV− and HIV+ men, it was significantly higher in HIV+/HAART− men than in HIV+/HAART+ men (p = 0.03); however, the latter two groups had similar prevalences of SDB (see table 4 and figure 1B). Similar results were obtained analyzing the AHI based on the AASM-recommended definition, except that the prevalence of SDB was significantly higher in HIV+/HAART− men than in HIV+/HAART+ men (p = 0.03; see Table S3).

**Table 4:** Prevalence and severity of SDB in Participants with BMI < 25 kg/m^2^.

	HIV –	All HIV+	P-value^a^	HIV+/HAART+	HIV+/HAART−	P-value^b^
	(N = 20)	(N = 51)		(N = 29)	(N = 22)	
RDI ≥5 events/h, n (%)	15 (75.0)	32 (62.8)	0.41	16 (55.2)	16 (72.7)	0.25
RDI (events/h)	8.2 (5.2–17.0)	9.9 (3.4–21.2)	0.83	7.7 (2.4–15.0)	15.5 (4.7–25.4)	0.02

RDI, respiratory disturbance index.

RDI is defined as the number of apneas and hypopneas (a ≥ 3% desaturation or an arousal) per hour of sleep.

Values shown are N(%) or median (25^th^ percentile–75^th^ percentile).

a P-values are for comparison of HIV− to HIV+ participants.

b P-values are for comparison of HIV+/HAART+ to HIV+/HAART− participants.

Comparisons of data represented by medians were performed using the Wilcoxon ranksum test for 2 group comparisons. Comparisons of categorical data represented by percent were performed using chi-square analysis and the Fisher’s exact test.

Second, we examined the associations of BMI, age, and race with SDB in multivariate models stratified by HIV status and HAART use. In these analyses, BMI remained significantly associated with the presence of SDB in the HIV− and HIV+/HAART+ men, but not in the HIV+/HAART− men (Table 5 and Table S4). The relation between age and SDB was no longer significant, despite the fact that age was independently associated with SDB in the multivariate analyses described above. This disparity may be due to the smaller group sizes in the stratified analyses.

**Table 5:** Adjusted odds ratios for associations of BMI and age with SDB at different RDI thresholds after stratification for HIV and HAART status*.

	N†	BMI (per kg/m^2^)	Age (per decade)
HIV− (N = 60)			
RDI ≥5 events/h	52	1.18 (0.98, 1.43)	1.95 (0.56, 6.72)
RDI ≥10 events/h	41	**1.32 (1.11, 1.57)**	2.05 (0.81, 5.18)
RDI ≥15 events/h	35	**1.43 (1.17, 1.75)**	2.08 (0.84, 5.16)
HIV+/HAART+ (N = 58)			
RDI ≥5 events/h	41	**1.25 (1.04, 1.50)**	1.46 (0.61, 3.50)
RDI ≥10 events/h	34	**1.24 (1.05, 1.46)**	1.05 (0.47, 2.32)
RDI ≥15 events/h	28	**1.30 (1.09, 1.54)**	2.15 (0.91, 5.09)
HIV+/HAART− (N = 41)			
RDI ≥5 events/h	30	0.93 (0.77, 1.13)	2.06 (0.76, 5.54)
RDI ≥10 events/h	22	0.92 (0.78, 1.10)	**2.72 (1.04, 7.15)**
RDI ≥15 events/h	18	0.94 (0.79, 1.11)	2.05 (0.85, 4.94)

*Adjusted for race.

† N, number of participants with SDB at the specified cutpoint.

RDI, respiratory disturbance index.

RDI is defined as the number of apneas and hypopneas (a ≥3% desaturation or an arousal) per hour of sleep.

Odds ratio and confidence intervals in bold represent observations that were statistically significant.

Note: When HIV+ men were combined into one group (N = 99), ORs for BMI and age were attenuated and of borderline significance. For example, for an RDI ≥5 events/h, BMI had OR: 1.10 [95% CI:0.97–1.25]] and age had OR 1.05 [95% CI: 0.99–1.12. Similar findings were observed for an RDI ≥10 and ≥15 events/h (data not shown).

We also examined the relations between lipohypertrophy and prevalence of SDB. In the entire study sample, after adjustment for age, BMI, race, HIV status, and HAART use, lipohypertrophy was significantly associated with SDB (odds ratio 3.68; 95% CI: 1.04–13.00) and with moderate-to-severe SDB (odds ratio 2.34; 95% CI: 0.94–5.80). In similar analyses stratified by HIV status, lipohypertrophy was significantly associated with moderate-to-severe SDB (odds ratio 3.72; 95% CI: 1.26–11.01) in HIV+ men, but not in HIV− men (odds ratio 0.40; 95% CI: 0.05–2.98). Similar associations were observed using the AHI based on the AASM-recommended definition.

### Fatigue, Sleepiness, and SDB

Fatigue was reported more commonly by HIV+ than HIV− men, both for three or more days (25.5% vs. 6.7%, respectively; p = 0.003) and for 2 or more weeks (17.2% vs. 5.0%, respectively; p = 0.03), especially for HIV+/HAART+ men (Table 6). Fatigue was less prevalent in men with than without SDB (16.4% vs. 25.0%, respectively; p = 0.24).

**Table 6:** Self-Reported Fatigue, Sleepiness, and Sleep Architecture by HIV and HAART status.

	HIV−	All HIV+	P-value^a^	HIV+/HAART+	HIV+/HAART−	P-value^b^
	(n = 60)	(n = 99)		(n = 58)	(n = 41)	
Fatigue ≥3 days, n (%)	4 (6.7)	25 (25.1)	0.003	18 (31.6)*	7 (17.1)	0.16
Fatigue ≥2 weeks, n (%)	3 (5.0)	17 (17.2)	0.03	12 (20.7)*	5 (12.2)	0.30
ESS score	8.0(5.5–11.5)	8.0(5.0–11.0)	0.18	8.0(5.0–11.0)	7.0(4.5–10.5)	0.83
ESS ≥11, n (%)	19 (31.7)	26 (26.5)	0.59	16 (27.6)	10 (25.0)	0.82
Sleep Parameters						
Total sleep time, min	403 (338–432)	402(365–434)	0.54	393 (372–427)	413 (361–445)	0.29
Time awake after sleep onset, min	64 (36–123)	71(34–97)	0.60	71 (44–97)	70 (28–94)	0.29
Time awake after sleep onset, % of time in bed	12.9 (7.4–25.1)	14.4(7.0–20.0)	0.65	14.5 (9.6–19.8)	14.4 (5.7–20.4)	0.47
Sleep latency, min	5.2 (2.0–9.2)	6.1(2.3–12.8)	0.35	5.5 (2.3–12.6)	7.5 (2.6–13.9)	0.34
Sleep efficiency, %	84.5 (71.0–90.5)	84.0(77.0–90.0)	0.62	84.5 (76.0–90.0)	84.0 (77.0–92.0)	0.47
Stage N1, %	20.3 (13.6–33.3)	22.5 (15.6–30.3)	0.59	23.5 (17.2–30.5)	21.1 (12.6–28.4)	0.06
Stage N2, %	55.0 (48.0–60.4)	55.4(47.7–62.6)	0.78	55.0 (46.7–62.2)	55.5 (48.0–62.7)	0.49
Stage N3, %	0.3 (0.0–3.3)	0.1(0.0–4.4)	0.87	0.4 (0.0–4.9)	0.0 (0.0–1.8)	0.19
Stage R, %	19.8 (13.2–24.4)	18.2(13.7–24.2)	0.87	17.2 (12.5–22.2)	20.1 (16.5–26.3)	0.06

ESS: Epworth Sleepiness Scale.

ESS (median, IQR).

Values shown are N(%) or median (25^th^ percentile –75^th^ percentile).

a P-values are for comparison of HIV− to HIV+ participants.

b P-values are for comparison of HIV+/HAART+ to HIV+/HAART− participants.

*P<0.05, compared to HIV−.

Comparisons of data represented by medians were performed using the Wilcoxon ranksum test for 2 group comparisons.

Comparisons of categorical data represented by percent were performed using chi-square analysis and the Fisher’s exact test.

Findings with regard to excessive sleepiness differed both qualitatively and quantitatively from those pertaining to fatigue. Unlike fatigue, excessive sleepiness was distributed similarly between HIV+ and HIV− men (ESS score ≥11 in 26.5% vs. 31.7%, respectively; p = 0.59; Table 6) and between HIV+ men using or not using HAART (27.6% vs. 25.0%, respectively; p = 0.82; Table 6). Excessive sleepiness, unlike fatigue, was significantly associated with SDB, being more common in men with moderate or severe SDB (ESS score: 9.2±4.6) than in those with mild SDB (ESS score: 6.7±4.3, p = 0.004). Excessive sleepiness was also significantly associated with an RDI ≥15 events/h (odds ratio 2.74; 95% CI: 1.31–5.73; p = 0.007), and this was independent of HIV or HAART status. In contrast, the association between sleepiness and SDB was not apparent using the AASM-recommended definition: in this case, the ESS was comparable between men with moderate-severe SDB and those with mild SDB, (ESS score: 8.7±4.7 vs. 8.7±5.1, respectively; p = 0.99). In addition, excessive sleepiness using the recommended definition was not significantly associated with an AHI ≥15 events/h (odds ratio 1.23; 95% CI: 0.59–2.55; p = 0.58). Further supporting the distinction between fatigue and sleepiness, only 20.5% of men reporting excessive sleepiness also reported fatigue, and men with and without fatigue had similar ESS scores (9.1±4.6 vs. 8.2±4.6, respectively; p = 0.37).

Sleep apnea syndrome, defined as an RDI ≥5 events/h and an ESS ≥11, was present in 35/159 (22.0%) of the entire study population. It was more common in the HIV− (n = 16/60; 26.7%) than the HIV+ men (19/99; 19.2%) (p = 0.32). Among HIV+ men, the HIV+/HAART+ group had a slightly higher prevalence of sleep apnea syndrome (14/58; 24.1%) than the HIV+/HAART− men (5/41; 12.2%), also not significant (p = 0.20). A similar prevalence of sleep apnea syndrome (mean 20.1%, range 9.8–24.1%) was observed using the AHI (AHI ≥5 events/h and an ESS ≥11), based on the AASM-recommended definition.

The above group differences in fatigue were not due to differences in sleep architecture, i.e., total sleep time, sleep efficiency, and distribution of sleep stages, which were virtually indistinguishable across the study groups (Table 6). However, all subject groups manifested high percentages of stage N1 sleep, with median values ranging from 20.3–23.3% compared to normative data (mean stage N1 sleep is ≈ 6% of total sleep time for adults between 45–55 years of age) [40]. When analyses were restricted to participants aged 45–55 years (n = 69), stage N1 sleep remained elevated across all 3 groups (18.4–28.6%).

### Comparisons of Sleepiness Between the Study Sample, the Parent Cohort, and the SHHS Sample

Given the high prevalence of SDB and sleep apnea syndrome observed in HIV− and HIV+ men, the Epworth Sleepiness scale (ESS) was administered post-hoc to participants at the Baltimore site of the MACS (n = 399), whether or not they had participated in the current study. Men who participated in the present study reported excessive sleepiness (ESS ≥11) more often than in those who did not participate (27% vs. 20%, respectively; odds ratio 1.81; 95% CI: 1.12–2.92). However, men who participated in the study were equally likely to participate whether they were HIV+ or HIV− (odds ratio for participation of HIV+ relative to HIV− men was 0.81; 95% CI: 0.51–1.31), indicating a non-differential bias. Furthermore, of the participants screened and found to be eligible for the study, there were similar rates of participation (i.e. final enrollment into the study) among the 3 groups (HIV−: 93.5%, HIV+/HAART+: 92.3%, HIV+/HAART−: 89.1%). Community participants from SHHS matched to HIV− men, described above, had similar ESS scores as the HIV− men (8.0 [IQR: 6.0–12.0] vs. 8.5 [IQR: 6.0–12.0], respectively).

## Discussion

There are several notable observations in the current study of men from the Multicenter AIDS Cohort Study (MACS). First, HIV− men demonstrated a higher prevalence and severity of SDB than HIV+ men, although this was not significant after adjustments for HAART use, BMI, age, and race. Second, the prevalence of SDB was higher than expected across all study groups, even in men of normal BMI. This was particularly true for the HIV+ men, who had prevalences of ∼70% despite significantly lower BMIs and younger age than the HIV− men. Second, lipohypertrophy in HIV+ men, but not in HIV− men, was independently associated with moderate- to-severe SDB. Third, consistent with previous reports [8]–[12], the frequency of fatigue was higher in HIV+ men than in HIV− men - a finding that was most prominent in HIV+/HAART+ group. Finally, excessive daytime sleepiness, but not fatigue, was significantly correlated with the moderate-severe SDB based on the AASM-alternative definition that includes arousals, compared to no association based on the AASM-recommended definition that does not include arousals.

### HIV Infection, HAART, and SDB

In community studies, 17–24% of overweight men had polysomnographic evidence of SDB (≥5 events/h), and 3–4% had the sleep apnea syndrome (defined as ≥5 or ≥10 events/hr with symptoms of daytime sleepiness) [1], [39]. In healthy, predominantly obese (mean BMI = 30.5 kg/m^2^), middle-aged men, the prevalence of SDB was higher at 62% in one study [41]. In the present study, although HIV+ men had prevalences of SDB and sleep apnea syndrome (72% and 19%, respectively) that were comparable to what is reported in moderate to severely obese men [41], [42], these prevalences were not significantly different from those of the HIV− men (87% and 27%, respectively). The higher prevalence of SDB in the HIV− men compared to HIV+ men may be partially explained by the higher BMI (28.6 vs. 25.4 kg/m^2^) and age (53.7 vs. 49.9 years old). However, HIV− men had higher prevalences and severity of SDB and oxygen desaturation than community-dwelling men from the Sleep Heart Health Study (SHHS) matched on age, BMI, and race. Taken together, these data indicate that the HIV− men studied from the MACS are not representative of the general population of men and are at higher risk for SDB. This higher risk could be due to known and unknown factors related to lifestyle or cohort membership that increased the prevalence of SDB for the entire cohort, regardless of HIV status. For example, use of illicit drugs (33–46% prevalences in the three study groups), hepatitis C infection (21–37%), and former and current smoking (72–84%) were factors that were elevated across the groups compared to the general population. The possibility that all MACS participants are at increased risk of SDB merits further investigation.

Although there was a higher prevalence of SDB in HIV− men, several observations suggest that HIV infection and/or HAART exposure may increase the prevalence of SDB and that this effect may have been obscured by the differences in BMI and age between HIV+ and HIV− men. First, among HIV+ men, lipohypertrophy – a common consequence of HAART exposure - was independently associated with moderate-to-severe SDB (OR 3.7; 95% CI: 1.0–11.0). Visceral adiposity is associated with an increased risk for SDB [23]. Second, the prevalence of SDB was significantly higher in the HIV+/HAART− men with prior HAART exposure than in those who were truly HAART-naive (90.0% vs. 57.1%; p = 0.02); this might be due to the persistence of HAART-related lipodystrophy after discontinuation of HAART [43]. However, there was no evidence that HAART use affected the severity of SDB, which may reflect opposing effects of HAART in stimulating visceral adiposity but also reducing HIV-mediated inflammation [15], [16]. Finally, in non-obese men, the median RDI was higher in the HIV+/HAART− men (15.5 events/h) than in the HIV+/HAART+ (7.7 events/h) and the HIV− (8.2 events/h) men; this finding was of borderline statistical significance (p = 0.06), possibly due to the smaller sample size. Although these observations must be interpreted cautiously, the possibility that HIV+ men have a higher prevalence of SDB at a lower BMI than HIV− men requires further investigation.

In general, the two primary mechanisms by which SDB develops are alterations in pharyngeal anatomy and blunted neuromuscular control of the upper airway [44]–[47]. HIV infection and HAART exposure both could contribute to both of these mechanisms. HIV infection may predispose to SDB either through adenotonsillar hypertrophy, which narrows the airway, [15], [16], but is uncommon since the advent of HAART, or through increased systemic production of somnogenic cytokines such as TNF-α and IL-1β [6], [48]–[50], which act through neural mechanisms [23]. We recently reported preliminary evidence that elevated plasma HIV viral loads and serum C-reactive protein concentrations were independently associated with moderate-to-severe SDB [51]. Further support for this inflammatory mechanism comes from the finding that etanercept, a TNF-α inhibitor, reduced RDI and sleepiness, and lowered serum IL-6 levels [52]. While this mechanism might have been operative in the HIV+/HAART− group, it is likely to be less important in the HIV+/HAART+ group, nearly all of whom had undetectable plasma HIV RNA concentrations. HAART-associated lipodystrophy, particularly visceral adiposity, may increase susceptibility for SDB through a narrowed pharyngeal airway [53], [54] **,** as was confirmed by computed tomography in a case report of one patient [15]. HAART-induced visceral adiposity is also associated with increased concentrations of inflammatory adipokines [55]–[61], which may depress the central nervous system and neuromuscular control of the upper airway [23], [24].

### Sleepiness, Fatigue, and Sleep architecture in HIV infection

Excessive sleepiness was roughly equally common (25–30%) in all three study groups. Men with moderate-to-severe SDB were more likely than men with mild SDB to have sleepiness. One notable finding was that excessive sleepiness was associated with SDB as defined by the RDI, but not as defined using the AHI. Since recurrent arousals are well known to result in sleepiness [31]–[34], this disparity most likely reflects the fact that the RDI includes respiratory-related arousals in its definition, while the AHI definition does not.

In contrast to sleepiness, fatigue in HIV+ men, particularly those using HAART, was not related to the presence or severity of SDB or to the presence of excessive sleepiness. These findings are consistent with previous reports [4], [5], [62]. The lack of correlation between fatigue and sleepiness supports the notion that these symptoms reflect distinct entities [5], [62]–[64]. Fatigue has been reported as a lack of energy or as tiredness after exercise that may lead to a period of inactivity. Sleepiness, on the other hand, represents a tendency to fall asleep [62] and depends on the duration of prior wakefulness. Taken together, our data suggest that clinicians caring for HIV-infected patients should carefully distinguish between these two symptoms in considering whether an individual has SDB, particularly when the individual is not obese.

Alterations in sleep architecture in HIV disease have been associated with increased circulating levels TNF-α and interleukin 1-beta (IL-1β) [6], [48], which have somnogenic effects that may perturb sleep and daytime function [49], [50], [65]–[68]. Our finding of similar sleep architecture in all study groups differed from previous reports, in which patients with early HIV infection had sleep fragmentation and altered sleep architecture which were associated with sleepiness [6], [69], [70].

### Limitations of the Current Study

The present study had several limitations. First, only men were studied, and the findings may not apply to women with or at risk for HIV infection. Second, as discussed, despite statistical adjustments, some effects of HIV infection or HAART on SDB may have been obscured by the differences in BMI and age between the HIV− and HIV+ men. For example, Pavlova et al. demonstrated that the prevalence of sleep apnea increases after the age of 50 years old in asymptomatic individuals [71]. Future studies should pay careful attention to the level of obesity and age of people enrolled. Third, the high prevalence of SDB in our sample might have been due to a selection bias. For example, the fact that eligible men were recruited from the parent study using mailed letters and flyers may have resulted in men with self-perceived sleepiness preferentially volunteering for the study. Selection bias is less likely to explain the high prevalence of SDB seen in HIV− and HIV+ men since there were similarly high levels of participation among those eligible across the three study groups. In addition, although participants in the present study were sleepier than in the parent cohort, they were equally likely to participate whether they were HIV+ or HIV−, indicating a non-differential bias. Furthermore, community participants from SHHS matched to HIV− men had similar ESS scores, suggesting that participants in the current study were no sleepier than a comparable sample of community dwelling men. Thus, selection bias is unlikely to explain the findings of this study. A fourth potential limitation, as noted, is that the HIV+/HAART− group included some men who had received HAART more than one year before this study, thus limiting our ability to separate the effects of HAART from those of HIV infection on the prevalence of SDB.

### Potential Implications

Further studies are needed to confirm and explain the high prevalence of SDB in the HIV+ men and HIV− men studied. In addition, given the potential effects of HIV infection and HAART on adiposity and inflammation, longitudinal studies are necessary to assess the effect of chronic HIV infection and HAART on the incidence and progression of SDB, and the mechanisms of these effects. Studies in women are also needed to examine whether there are similar observations regarding HIV infection, HAART exposure, and SDB. For the present, clinicians caring for HIV-infected people should consider SDB as an etiology for sleepiness, even in men of normal weight.

## Supporting Information

Table S1 Prevalence and severity of SDB, stratified by HIV status and HAART use.
(DOC)

Table S2 Association of SDB with HIV status, BMI, age, and race.
(DOC)

Table S3 Prevalence and severity of SDB in Participants with BMI <25 kg/m^2^.
(DOC)

Table S4 Adjusted odds ratios for associations of BMI and age with SDB at different AHI thresholds after stratification for HIV and HAART status^*^.
(DOC)
