# Supplementary material for: Sleep Disordered Breathing, Fatigue, and Sleepiness in HIV-Infected and -Uninfected Men
Source: PLoS One. 2014 Jul 3;9(7):e99258. doi: 10.1371/journal.pone.0099258 (PMC4084642; doi:10.1371/journal.pone.0099258)
Supplement: Table S1 — Prevalence and severity of SDB, stratified by HIV status and HAART use. (DOC) [file pone.0099258.s001.doc]

**Table S1 - Prevalence and Severity of Sleep Disordered Breathing**, stratified by HIV status and HAART use

|  |  | **HIV–** | **All HIV+** | **P-value^a^** | **HIV+/HAART+** | **HIV+/HAART-** | **P-value^b^** |
| --- | --- | --- | --- | --- | --- | --- | --- |
|  |  | **(N=60)** | **(N=99)** |  | **(N=58)** | **(N=41)** |  |
| 1. **Prevalence** |  |  |  |  |  |  |  |
|  |  |  |  |  |  |  |  |
| Prevalence By Apnea Hypopnea Index (AHI)^c^ |  |  |  |  |  |  |  |
| AHI ≥ 5 events/h |  | 42 (70.0) | 54 (54.6) | 0.07 | 32 (55.2) | 22 (53.7) | 1.00 |
| AHI ≥ 10 events/h |  | 30 (50.0) | 36 (36.7) | 0.10 | 21 (36.2) | 15 (36.6) | 1.00 |
| AHI ≥ 15 events/h |  | 23 (38.2) | 28 (28.3) | 0.22 | 15 (25.9) | 13 (31.7) | 0.65 |
|  |  |  |  |  |  |  |  |
| Prevalence By Severity (events/h) |  |  |  |  |  |  |  |
| Normal (< 5.0) |  | 18 (30.0) | 45 (45.5) | 0.03 | 26 (44.8) | 19 (46.3) | 0.83 |
| Mild (5.0 -14.9) |  | 19 (31.7) | 26 (26.3) |  | 17 (29.3) | 9 (22.0) |  |
| Moderate (15.0 – 29.9) |  | 10 (16.7) | 21 (21.2) |  | 11 (19.0) | 10 (24.3) |  |
| Severe (≥ 30.0) |  | 13 (22.2) | 7 (7.1) |  | 4 (6.0) | 3 (7.3) |  |
|  |  |  |  |  |  |  |  |
| 1. **AHI Distribution** |  |  |  |  |  |  |  |
|  |  |  |  |  |  |  |  |
| AHI (events/h)^a^ |  |  |  |  |  |  |  |
| NREM |  | 7.4 (2.1 – 23.1) | 4.3 (1.1 – 14.8) | 0.04 | 5.4 (1.0 – 14.8) | 3.7 (1.2 – 12.1) | 0.86 |
| REM |  | 11.5 (5.3 – 30.0) | 8.4 (2.6 – 23.9) | 0.18 | 8.2 (2.3 – 17.9) | 10.1 (4.4 – 24.9) | 0.51 |
| Total |  | 10.2 (3.8 – 25.1) | 5.9 (2.1 – 16.4) | 0.01 | 7.6 (2.1 – 16.4) | 5.2 (2.1 – 16.3) | **0.97** |
|  |  |  |  |  |  |  |  |
| Values shown are N(%) or median (25^th^ percentile – 75^th^ percentile) | | | | | | | |
| ^a^P-values are for comparison of HIV- to HIV+ participants | | | | | | | |
| ^b^P-values are for comparison of HIV+/HAART+ to HIV+/HAART- participants | | | | | | | |
| ^c^AHI is defined as the number of apneas and hypopneas (associated with a ≥ 4% desaturation) per hour of sleep | | | | | | | |
| Comparisons of data represented by medians were performed using the Wilcoxon ranksum test for 2 group comparisons. | | | | | | | |
| Comparisons of categorical data represented by percent were performed using chi-square analysis and the Fisher’s exact test. | | | | | | | |
