# Supplementary material for: Sleep Disordered Breathing, Fatigue, and Sleepiness in HIV-Infected and -Uninfected Men
Source: PLoS One. 2014 Jul 3;9(7):e99258. doi: 10.1371/journal.pone.0099258 (PMC4084642; doi:10.1371/journal.pone.0099258)
Supplement: Table S2 — Association of SDB with HIV status, BMI, age, and race. (DOC) [file pone.0099258.s002.doc]

**Table S2 – Association of SDB with HIV status, BMI, age, and race**

|  | **Unadjusted** | **Adjusted** |
| --- | --- | --- |
|  |  |  |
| HIV+^*^ | **0.51 (0.26 – 1.01)** | 0.89 (0.41 – 1.91) |
|  |  |  |
| BMI (kg/m^2^) | **1.19 (1.09,1.30)** | **1.19 (1.09, 1.30)** |
| Age (per decade) | **1.63 (1.10, 2.41)** | **1.73 (1.08, 2.78)** |
| Race^†^ | 0.96 (0.51, 1.83) | 1.21 (0.55, 2.65) |
| Similar results were obtained modeling an AHI ≥ 10 and an AHI ≥ 15 events/h. | | |
| Odd ratio (95% confidence interval) | | |
| ^*^Reference group: HIV- | | |
| Note: Results did not change appreciably when comparing HIV+/HAART+ (unadjusted OR: 0.53 [95% CI: 0.25 – 1.12]; adjusted OR: 0.88 [95% CI: 0.38 – 2.06]) and HIV+/HAART- men(unadjusted OR: 0.50 [95% CI: 0.22 – 0.1.13]; adjusted OR: 0.89 [95% CI: 0.36 – 2.25]) to HIV- men. | | |
