# Supplementary material for: Sleep Disordered Breathing, Fatigue, and Sleepiness in HIV-Infected and -Uninfected Men
Source: PLoS One. 2014 Jul 3;9(7):e99258. doi: 10.1371/journal.pone.0099258 (PMC4084642; doi:10.1371/journal.pone.0099258)
Supplement: Table S3 — Prevalence and severity of SDB in Participants with BMI <25 kg/m2. (DOC) [file pone.0099258.s003.doc]

**Table S3 – Prevalence and severity of SDB in Participants with BMI < 25 kg/m^2^**

|  |  | **HIV–** | **All HIV+** | **P-value^a^** | **HIV+/HAART+** | **HIV+/HAART-** | **P-value^b^** |
| --- | --- | --- | --- | --- | --- | --- | --- |
|  |  | **(N=20)** | **(N=99)** |  | **(N=29)** | **(N=22)** |  |
|  |  |  |  |  |  |  |  |
| AHI ≥ 5 events/h, n(%) |  | 7 (35.0) | 23 (45.1) | 0.59 | 14 (31.0) | 14 (63.6) | 0.03 |
|  |  |  |  |  |  |  |  |
| AHI (events/h) |  | 3.7 (1.8 – 8.9) | 4.1 (1.2 – 9.3) | 0.88 | 2.5 (0.9 – 7.6) | 5.7 (2.0 – 17.2) | 0.03 |
|  |  |  |  |  |  |  |  |
| AHI, apnea hypopnea index | | | | | | | |
| AHI is defined as the number of apneas and hypopneas (a ≥ 4% desaturation) per hour of sleep | | | | | | | |
| Values shown are N(%) or median (25^th^ percentile – 75^th^ percentile) | | | | | | | |
| ^a^P-values are for comparison of HIV- to HIV+ participants | | | | | | | |
| ^b^P-values are for comparison of HIV+/HAART+ to HIV+/HAART- participants | | | | | | | |
| Comparisons of data represented by medians were performed using the Wilcoxon ranksum test for 2 group comparisons. | | | | | | | |
| Comparisons of categorical data represented by percent performed using chi-square analysis and the Fisher’s exact test. | | | | | | | |
