# Supplementary material for: Sleep Disordered Breathing, Fatigue, and Sleepiness in HIV-Infected and -Uninfected Men
Source: PLoS One. 2014 Jul 3;9(7):e99258. doi: 10.1371/journal.pone.0099258 (PMC4084642; doi:10.1371/journal.pone.0099258)
Supplement: Table S4 — Adjusted odds ratios for associations of BMI and age with SDB at different AHI thresholds after stratification for HIV and HAART status*. (DOC) [file pone.0099258.s004.doc]

| **Table S4 – Adjusted odds ratios for associations of BMI and age with SDB at different AHI thresholds after stratification for HIV and HAART status^*^** | | | |
| --- | --- | --- | --- |
|  | N^†^ | BMI (per kg/m^2^) | Age (per decade) |
| HIV- (N=60) |  |  |  |
| AHI ≥ 5 events/h | 42 | **1.33 (1.11 – 1.59)** | 2.05 (0.79 – 5.29) |
| AHI ≥ 10 events/h | 30 | **1.41 (1.17 – 1.71)** | 1.67 (0.72 – 3.86) |
| AHI ≥ 15 events/h | 23 | **1.30 (1.11 – 1.53)** | 1.71 (0.79 – 3.69) |
|  |  |  |  |
| HIV+/HAART+ (N=58) |  |  |  |
| AHI ≥ 5 events/h | 32 | **1.35 (1.12 – 1.63)** | 1.56 (0.68 – 3.58) |
| AHI ≥ 10 events/h | 21 | **1.42 (1.16 – 1.76)** | **3.16 (1.14 – 8.73)** |
| AHI ≥ 15 events/h | 15 | **1.38 (1.13 – 1.68)** | 1.75 (0.64 – 4.81) |
|  |  |  |  |
| HIV+/HAART- (N=41) |  |  |  |
| AHI ≥ 5 events/h | 22 | 0.88 (0.72 – 1.06) | 2.29 (0.90 – 5.82) |
| AHI ≥ 10 events/h | 15 | 0.97 (0.81 – 1.16) | 2.27 (0.87 – 5.93) |
| AHI ≥ 15 events/h | 13 | 0.84 (0.67 – 1.06) | 2.51 (0.89 – 7.05) |
|  |  |  |  |
| ^*^Adjusted for race | | | |
| **^†^**N, number of participants with SDB at the specified cutpoint | | | |
| AHI, apnea-hypopnea index | | | |
| AHI is defined as the number of apneas and hypopneas (associated with a ≥ 4% desaturation) per hour of sleep | | | |
| Odds ratio and confidence intervals in bold represent observations that were statistically significant | | | |
| Note: When HIV+ men were combined into one group (N=99), OR’s for BMI and age were attenuated and no longer significant. For example, for an AHI ≥ 5 events/h, BMI had an OR 1.11 [95% CI:0.99 – 1.23] and age had an OR 1.05 [95% CI: 0.99 – 1.11]). Similar findings were observed for an AHI ≥ 10 and ≥ 15 events/h (data not shown). | | | |
